# Association of aldosterone excess and apnea-hypopnea index in patients with resistant hypertension

**DOI:** 10.1038/srep45241

**Published:** 2017-03-22

**Authors:** Xiao Ke, Wenyu Guo, Hu Peng, Chengheng Hu, Henghong Zhang, Changnong Peng, Xiaoqing Wang

**Affiliations:** 1 Department of Cardiology, Shenzhen Sun Yat-sen Cardiovascular Hospital, Shenzhen, China; 2 Department of Cardiology, the Fifth subsidiary Sun Yat-sen University Hospital, Zhuhai, China; 3 Department of Cardiology, the First Affiliated Hospital of Sun Yat-sen University, Guangzhou, China; 4 The Traditional Chinese Medicine Hospital of Jiu Jiang city, Jiu Jiang, China

## Abstract

The present study was to investigate the association of aldosterone excess and apnea-hypopnea index (AHI) in patients with resistant hypertension. Patients with resistant hypertension were enrolled and baseline characteristics including plasma aldosterone concentration (PAC) and 24 h-urine aldosterone levels were collected and compared between groups with different degrees of AHI as assessed by polysomnography. Association of key variables and AHI was then evaluated by univariate and multiple linear regression analysis. A total of 534 patients with resistant hypertension were enrolled and mean age was 57 ± 11 years. Overall, mean number of AHI was 21.7 ± 9.6 and nearly 92.3% of resistant hypertensive patients had obstructive sleep apnea (OSA). Mean PAC and 24 h-urine aldosterone level was 12.4 ± 6.3 ng/dL and 13.1 ± 6.8 ug, respectively. Compared with other groups, participants in the severe OSA group (AHI ≥ 30) had significantly higher PAC and 24 h-urine aldosterone level. Multiple linear regression analysis showed that PAC and 24 h-urine aldosterone levels were positively associated with AHI, while spironolactone was negatively associated with AHI, independent of age, gender, body mass index, smoking, plasma renin activity and diuretics. OSA is highly prevalent in patients with resistant hypertension and both PAC and 24 h-urine aldosterone level are significantly associated with AHI.

Essential hypertension is a major public health problem around the world and lowering blood pressure (BP) is beneficial for reducing the incidence of cardiovascular and kidney diseases^1^,^2^,^3^. Resistant hypertension, which is defined as clinic BP above 140/90 mm Hg despite using ≥3 anti-hypertensive medications including one diuretic at their optimal doses^4^, has a particularly poor prognosis owing to its persistent BP elevation and detrimental effects on cardiovascular system^5^. Therefore, it is clinically relevant to identify the underlying mechanisms associated with resistant hypertension so as to design an efficient therapeutic strategy to effectively lower BP in these extremely high-risk populations.

Previously, a substantial number of observational researches have shown that patients with hypertension and OSA were more difficult to have their BP control than hypertensive patients without OSA^6^,^7^, and the underlying mechanisms might be partly attributed to intermittent hypoxemia-induced renin-angiotensin-aldosterone system activation^8^. Increased plasma aldosterone concentration (PAC) subsequently resulted in sodium and fluid accumulation in neck tissue during sleep which caused upper airway obstruction and OSA^9^,^10^. Nevertheless, whether there is an independent relationship of PAC and 24 h-urine aldosterone level with the severity of OSA as indexed by the apnea-hypopnea index (AHI) has not been fully addressed. Whether this relationship is independent of diuretic or spironolactone therapy is also unknown. Furthermore, spironolactone has been recommended as the preferred fourth-line anti-hypertensive agent in patients with resistant hypertension^11^, data on the usage of spironolactone in our routine clinical practice is also lacking.

We therefore conducted a cross-sectional research to investigate the above clinically relevant issues. Our preliminary data show that OSA is highly prevalent in patients with resistant hypertension and both PAC and 24 h-urine aldosterone level are independently associated with OSA severity as indexed by the AHI. The application of aldosterone antagonist in our daily clinical practice is still extremely low, and in the future we should make concerted efforts to narrow the gap in order to improve BP control in these high-risk populations.

## Methods

### Studied subjects enrollment

The present research was approved by the clinical research ethical committee of Shenzhen Sun Yat-sen Cardiovascular Hospital. All the performances were in accordance to the Declaration of Helsinki. Informed consent was obtained from all participants. Included criteria were as follows: 45–75 years-old hypertensive patients; clinic BP ≥ 140/90 mm Hg despite treating with 3 anti-hypertensive medications including one diuretic at optimal doses for at least 4 weeks^4^; willing to have OSA evaluation by polysomnography and willing have biochemical measurements in a hospitalized setting. Excluded criteria were as follows: documented secondary hypertension; a previous diagnosis of OSA and/or had been treated with continuous positive airway pressure or devices; a previous medical history of myocardial infarction, ischemic/hemorrhagic stroke, heart failure or chronic kidney disease with estimated glomerular filtration rate (GFR) ≤ 60 ml/min/1.73 m^2^ using the Modification of Diet in Renal Disease formula^12^.

### Clinical data collection

Clinical data were collected using constructed questionnaire which included information about the demographic, anthropometric and medical history of all participants. Briefly, fasting venous blood were used to measure serum levels of fasting plasma glucose (FPG), total cholesterol (TC), potassium (K^+^), sodium (Na^+^), albumin (ALB), creatinine (Cr), blood urine nitrogen (BUN), plasma aldosterone concentration (PAC) and plasma renin activity (PRA). Aldosterone/renin ratio (ARR) was then calculated by the standard formula of PAC divided by PRA^13^. Hospitalized patients were helped by the charged nurses to collect 24 h urine after the first morning void was discarded. Twenty-four hour urine was collected to evaluate 24 h-urine aldosterone, 24 h-urine K^+^, 24 h-urine Na^+^ and 24 h-urine Cr levels by mass spectrometry^14^.

### Evaluation of OSA severity

Overnight PSG was performed in all participants using Philips Respironics Alice PDx. Thoraco-abdominal movements were monitored, and oxyhemoglobin saturation (SaO_2_) was also continuously monitored by a pulse oximeter and the mean and lowest SaO_2_ during sleep were recorded. Those with central sleep apnea were excluded and only those with OSA were included into final analysis. According to the current AASM guideline^15^, airflow complete blockage for more than 10 seconds, or >50% reduction in respiratory airflow accompanying >3% reduction in SaO_2_ for more than 10 seconds would register as apnea or hypopnea events, and the AHI was calculated by the total number of apnea and hypopnea events per sleep hour, with AHI of 5–14 were defined as mild, 15–29 moderate, and 30 or more severe OSA, respectively^15^.

### Statistical analysis

Continuous variables were presented as mean ± SD and categorical variables were presented by number and percentages of categories. The statistical significance of differences was analyzed by using one-way ANOVA for continuous variables and the chi-square or Fisher exact test for categorical variables. Pearson correlation analysis was used to evaluate simple linear relationship between AHI and other variables. Multivariate linear regression analysis was performed to evaluate the independent relationship between the AHI and other covariates, and the models were adjusted for potential confounding factors with stepwise strategies. Statistical analyze were computed using SPSS 17.0 (SPSS Inc, Chicago, IL). All statistical tests were two-sided and considered statistically significant when P < 0.05.

## Results

### General characteristics of participants

From January of 2014 to June of 2016, a total of 536 resistant hypertensive patients were enrolled from outpatient department and 2 patients subsequently diagnosed as central sleep apnea by polysomnography were excluded, and 534 participants were included into final analysis. As shown in Table 1, mean age was 57 ± 11 years and males accounted for 67.9% (n = 363). Mean clinic systolic/diastolic BP (SBP/DBP) were 159 ± 13 mm Hg and 98 ± 7 mm Hg respectively and mean number of current anti-hypertensive medication usage was 3.6 ± 0.4. Body mass index (BMI) was 24.7 ± 5.4 Kg/m^2^ and proportion of obesity (defined as BMI ≥ 30 kg/m^2^ 16) was 14.8%. Based on polysomnography findings, mean number of the AHI was 21.7 ± 9.6 and nearly 92.3% of resistant hypertensive patients had OSA (n = 493). Mean PAC and 24 h-urine aldosterone level was 12.4 ± 6.3 ng/dL and 13.1 ± 6.8 ug, respectively.

### Comparisons by categories of OSA severity

On the basis of AHI, all participants were classified into without OSA, and mild, moderate and severe OSA groups. As shown in Table 2, compared with other groups, the participants in the severe OSA group were more likely males and had higher values of BMI and neck girth, with corresponding higher prevalence of obesity. In addition, SBP was also significantly higher in the severe OSA group. With respect to biochemical parameters, PAC and ARR were all significantly higher in the severe OSA group. Corresponding urinary findings in terms of higher 24 h-urine potassium and 24 h-urine aldosterone levels and lower 24 h-urine sodium level were also observed in the severe OSA group. Compared with other groups, mean number of apnea-hypopnea events per sleep hour was also significantly higher in the severe OSA group, with corresponding lower mean and lowest SO_2_ levels.

### Anti-hypertensive medications

Current anti-hypertensive medications usages were evaluated and compared. As shown in Table 3, no significant difference in each specific anti-hypertensive drug was observed among each group. Notably, a large proportion of participants were treating with angiotensin converting enzyme inhibitor/angiotensin receptor blocker (ACEI/ARB), calcium channel blocker and diuretic, while the percentage of patients treating with spironolactone in each group was less than 15%.

### Univariate and multiple linear regression analysis

Univariate regression analysis was performed to evaluate the independent relationship between AHI and key covariates. As shown in the Table 4, age, male gender, BMI, increased neck girth, PAC, 24 h-urine aldosterone level and spironolactone were all independently associated with the AHI. In the multiple linear regression analysis, parameters with independent association with AHI were included and underwent stepwise adjustment. As shown in the Table 5, from model 1 to model 3, it showed that neck girth, PAC and 24 h-urine aldosterone level were positively associated with AHI and usage of spironolactone was negatively associated with the AHI, which were independent of age, male gender, BMI, smoking status, plasma renin activity, diuretic and ACEI/ARB usage.

## Discussion

Our current cross-sectional research has several principal findings: first, in resistant hypertensive patients, both PAC and 24 h-urine aldosterone levels are positively associated with the AHI; second, spironolactone but not furosemide or hydrochlorothiazide is negatively associated with the AHI; third, the percentage of spironolactone usage in patients with resistant hypertensive in our routine clinical practice is extremely low.

Similar to previous findings^6^,^8^,^17^, our current study also showed that there was a high prevalence of unrecognized OSA in patients with resistant hypertension. For example, Logan *et al*. enrolled 41 drug-resistant hypertensive patients and they reported that the prevalence of OSA was 83%^17^. While in our much larger sample size study, we found that nearly 92.3% of resistant hypertensive participants had OSA. However, in the previous study^17^, there was only 85% participants were treating with diuretic, while in our current study, enrolled participants were treating with 3 different classics of BP-lowering drugs including one diuretic at optimal doses, which might avoid recruiting patients with suboptimal treatment. In a case-control study^6^, Gonc alves *et al*. reported that compared to the control group, OSA prevalence was significantly higher in the resistant hypertensive group (71% versus 38%) and OSA was independently associated with resistant hypertension. Notably, diagnostic criterion of OSA was based on the AHI ≥ 10^6^. While in our current study, in concert with the AASM guideline recommendation^15^, OSA was diagnosed when the AHI was ≥5 and this discrepancy in diagnostic criterion might partly attribute to the difference in OSA prevalence between different studies. In a milestone study^8^, Pedrosa and colleagues enrolled 125 resistant hypertensive patients and they used a much stricter criterion to diagnose OSA, that is AHI ≥ 15, they found that OSA was the most common cause of resistant hypertension with a prevalence of 64%. However, if they used AHI ≥ 5 to define OSA, it was probable that OSA prevalence in resistant hypertensive patients would be even higher. Nevertheless, these findings together supported the notion that OSA might be the most significant cause of resistant hypertension and patients with difficult BP control despite treating with 3 or more BP lowering drugs should routinely undergo OSA evaluation^18^.

Although the relationship between OSA and resistant hypertension has been extensively evaluated, the associated mechanisms were still far from clear. Previously, some studies have revealed the relationship of PAC with OSA severity and BP level. For example, in a cross-sectional study^19^, Gonzaga *et al*. enrolled 109 resistant hypertensive patients, and they found that OSA severity was significantly associated with PAC. Similarly, we also observed that there was a trend, that is, the more severe OSA, the higher PAC and 24 h-urine aldosterone levels as indicated in Table 2. We additionally showed that, independent of potential confounders such as BMI, neck girth, diuretic and aldosterone antagonist, both PAC and 24 h-urine aldosterone level were positively associated with the AHI in a multiple linear regression model as presented in Table 5. While in the previous study, Gonzaga *et al*. did not evaluate whether the significant relationship between OSA severity and PAC was independent of other potential confounding factors^19^. Recently, Dudenbostel and colleagues reported that in patients with resistant hypertension, BMI was associated with 24 hour urinary aldosterone level, and obesity might contribute to the excess of aldosterone^13^. In our current study, we also observed that both BMI and obesity prevalence in the severe OSA group were significantly higher than the other groups. Therefore, it could be possible that obesity-induced PAC elevation might play an important role between resistant hypertension and OSA^20^. Indeed, the relationship between OSA and hypertension was bidirectional, and obesity and aldosterone excess were the most important mediators as indicated^21^.

Aldosterone antagonist such as spironolactone has been recognized as the preferred add-on therapy for BP-lowering in resistant hypertension since the publication of the PATHYWAY-2 clinical trial^11^. Recently, Hwang *et al*. conducted a study to investigate the trend of antihypertensive medication use among US patients with resistant hypertension from 2008 to 2014^22^, and they found that despite advocacy of aldosterone antagonist usage in resistant hypertension, aldosterone antagonist application in clinical practice was increased only modestly (from 7.3% to 10.2%). Similarly, in our current study, we found that less than 15% of patients with resistant hypertension were treating with spironolactone. These data was essential as to showing to us how big the gap was between the guideline recommendation and our daily clinical practice. To our knowledge, increasing spironolactone prescription might be a convenient and cost-effective approach by which we could narrow the gap and improve BP control.

Notably, in the multiple linear regression analysis, after adjusted for potential confounding factors, we observed that only spironolactone was negatively associated with the AHI, and the underlying mechanisms might be multiple folds. On the one hand, it was noted that fluid retention was one of the major mechanisms of resistant hypertension, and during sleep time, rostral shift from lower extremity could lead to peri-pharyngeal fluid accumulation which in turn caused upper airway narrowing and OSA development^23^,^24^,^25^. Knowingly, spironolactone treatment was beneficial for increasing sodium and fluid excretion which in turn might help to improve upper airway obstruction and reduce the AHI. On the other hand, intermittent hypoxemia induced renin-angiotensin-aldosterone (RAA) activation which could also result in increased sodium and fluid retention^23^,^26^. Therefore, spironolactone therapy might also reduce the AHI through counteracting these unfavorable effects. Indeed, in the univariate regression analysis, no significant association was observed between the AHI and ACEI/ARB treatment which might also indicate that the effects of RAA activation on OSA were related to aldosterone-induced fluid retention.

There were several limitations of our current study. First, the inherent limitation of cross-sectional design could not allow us to draw causal relationship between increased PAC and the AHI, although a positive association was observed in a multiple linear regression analysis. Second, a small proportion of patients were treating with spironolactone when PAC and 24 h-urine aldosterone level were measured which might influence the relationship between PAC and the AHI. However, in the multiple linear regression analysis, we observed that both PAC and 24 h-urine aldosterone level were significantly associated with the AHI, which was independent of spironolactone usage. Moreover, regarding the ethical issues, it might be inappropriate for us to discontinue anti-hypertensive medications. Finally, participants in our current study were relatively healthy with respect to without existing overt cardiovascular or kidney disease and low prevalence of obesity and diabetes. Therefore, the data should not be extrapolated to other more morbid populations.

In conclusion, our current study showed that OSA is highly prevalent in patients with resistant hypertension, and both PAC and 24 h-urine aldosterone level are significantly associated with the AHI. The application of aldosterone antagonist is still extremely low and clinical trials evaluating the impact of adding spironolactone on top of ACEI/ARB, CCB and diuretic therapy may represent important future research steps toward improving BP control rate in patients with resistant hypertension.

## Additional Information

**How to cite this article**: Ke, X. *et al*. Association of aldosterone excess and apnea-hypopnea index in patients with resistant hypertension. *Sci. Rep.* **7**, 45241; doi: 10.1038/srep45241 (2017).

**Publisher's note:** Springer Nature remains neutral with regard to jurisdictional claims in published maps and institutional affiliations.

## Figures and Tables

**Table 1:** General characteristics.

Variables	Values
Age (years)	57 ± 11
Male, n (%)	363 (67.9)
Body mass index (kg/m^2^)	24.7 ± 5.4
Obesity, n (%)	79 (14.8)
Neck girth (cm)	37.8 ± 5.4
Current smoking, n (%)	247 (46.3)
Diabetes mellitus, n (%)	98 (18.4)
eGFR (ml/min/1.73 m^2^)	94.5 ± 10.6
SBP (mm Hg)	159 ± 13
DBP (mm Hg)	98 ± 7
FPG (mmol/L)	6.1 ± 0.4
TC (mmol/L)	5.0 ± 0.5
K^+^ (mEq/L)	3.9 ± 0.4
Na^+^ (mEq/L)	143 ± 6
ALB (g/L)	43 ± 5
Cr (umol/L)	117 ± 16
BUN (mmol/L)	7.1 ± 2.4
PAC (ng/dL)	12.4 ± 6.3
PRA(ng/mL/h)	4.2 ± 2.9
ARR	8.7 ± 3.5
24 h-urine K^+^ (mEq)	65.4 ± 28.7
24 h-urine Na^+^ (mEq)	168.3 ± 55.7
24 h-urine aldosterone (ug)	13.1 ± 6.8
24 h-urine Cr (mg)	1547 ± 434
AHI (number/hour)	21.7 ± 9.6
OSA, n (%)	493 (92.3)
Mean SO_2_ (%)	94.7 ± 0.6
Lowest SO_2_ (%)	86.4 ± 1.9
Anti-hypertensive agents (n)	3.6 ± 0.4

Denote: eGFR = estimated glomerular filtration rate; SBP = systolic blood pressure; DBP = diastolic blood pressure; FPG = fasting plasma glucose; TC = total cholesterol; ALB = albumin; Cr = creatinine; BUN = blood urine nitrogen; PAC = plasma aldosterone concentration; PRA = plasma renin activity; ARR = aldosterone-renin ratio; AHI = apnea hypopnea index; OSA = obstructive sleep apneal.

**Table 2:** Comparisons by categories of OSA severity.

Variables	Without OSA (n = 41)	Mild (n = 126)	Moderate (n = 167)	Severe (n = 200)
Age (years)	59 ± 8	56 ± 10	57 ± 10	57 ± 9
Male, n (%)	22 (53.7)	73 (57.9)	105 (62.9)	163 (81.5)*
Body mass index (kg/m^2^)	21.4 ± 2.0	22.9 ± 2.5	24.0 ± 3.2	25.3 ± 3.9*
Obesity, n (%)	4 (9.8)	16 (12.7)	25 (14.9)	34 (17.0)*
Neck girth(cm)	32.6 ± 3.1	34.4 ± 5.0	37.1 ± 3.9	38.6 ± 4.3*
Current smoking, n (%)	18 (43.9)	64 (50.8)	72 (43.1)	93 (46.5)
Diabetes mellitus, n (%)	8 (19.5)	22 (17.5)	31 (18.6)	37 (18.5)
eGFR (ml/min/1.73 m^2^)	98.3 ± 10.2	95.4 ± 8.9	96.3 ± 11.2	97.2 ± 9.5
SBP (mm Hg)	150 ± 8	154 ± 11	158 ± 13	163 ± 10*
DBP (mm Hg)	96 ± 6	98 ± 7	100 ± 8	99 ± 7
FPG (mmol/L)	6.0 ± 0.5	6.2 ± 0.6	6.1 ± 0.5	6.0 ± 0.4
TC (mmol/L)	5.0 ± 0.4	5.2 ± 0.5	5.1 ± 0.4	5.2 ± 0.6
K^+^ (mEq/L)	4.0 ± 0.3	4.0 ± 0.5	3.9 ± 0.3	3.8 ± 0.3
Na^+^ (mEq/L)	143 ± 8	142 ± 6	144 ± 5	146 ± 8
ALB (g/L)	45 ± 4	43 ± 6	42 ± 5	43 ± 4
Cr (umol/L)	120 ± 12	113 ± 10	118 ± 11	116 ± 9
BUN (mmol/L)	7.3 ± 2.2	7.1 ± 2.6	7.2 ± 2.5	7.1 ± 2.0
PAC (ng/dL)	9.8 ± 4.2	10.8 ± 5.6	12.9 ± 6.2	14.3 ± 7.2*
PRA (ng/mL/h)	4.3 ± 2.7	4.1 ± 2.6	4.3 ± 2.3	4.4 ± 3.0
ARR	5.6 ± 1.2	7.8 ± 2.1	8.9 ± 2.8	10.1 ± 3.6*
24 h-urine K^+^ (mEq)	48.7 ± 19.5	57.6 ± 20.4	66.3 ± 25.7	69.3 ± 26.2*
24 h-urine Na+ (mEq)	173.6 ± 60.2	170.4 ± 53.9	163.4 ± 49.8	159.5 ± 48.8*
24 h-urine aldosterone (ug)	8.6 ± 3.5	10.2 ± 4.1	12.9 ± 5.4	14.5 ± 6.6*
24 h-urine Cr (mg)	1496 ± 356	1589 ± 403	1507 ± 532	1563 ± 462
AHI (number/hour)	3.5 ± 1.1	10.6 ± 2.9	22.8 ± 5.6	35.3 ± 4.2*
Mean SO_2_ (%)	98.6 ± 0.5	96.2 ± 0.7	94.2 ± 0.5	92.3 ± 0.5*
Lowest SO_2_ (%)	92.6 ± 1.5	89.3 ± 1.2	86.0 ± 1.4	85.2 ± 1.5*
Anti-hypertensive agents (n)	3.6 ± 0.4	3.5 ± 0.4	3.6 ± 0.5	3.5 ± 0.5

Denote: *P < 0.05 versus other groups; eGFR = estimated glomerular filtration rate; SBP = systolic blood pressure; DBP = diastolic blood pressure; FPG = fasting plasma glucose; TC = total cholesterol; ALB = albumin; Cr = creatinine; BUN = blood urine nitrogen; PAC = plasma aldosterone concentration; PRA = plasma renin activity; ARR = aldosterone-renin ratio; AHI = apnea hypopnea index; OSA = obstructive sleep apneal.

**Table 3:** Anti-hypertensive medications.

Medications	Without OSA (n = 41)	Mild (n = 126)	Moderate (n = 167)	Severe (n = 200)
ACEI/ARB, n (%)	36 (87.8)	109 (86.5)	144 (86.2)	173 (86.5)
CCB, n (%)	31 (75.6)	97 (76.9)	124 (74.3)	147 (73.5)
Diuretic, n (%)	41 (100)	126 (100)	167 (100)	200 (100)
Spironolactone, n (%)	6 (14.6)	17 (13.5)	24 (14.4)	18 (14.0)
Beta-blocker, n (%)	15 (36.6)	45 (35.7)	65 (38.9)	68 (34.0)
Alpha-blocker, n (%)	13 (31.7)	42 (33.3)	54 (32.3)	64 (32.0)

**Denote**: ACEI = angiotensin converting enzyme inhibitor; ARB = angiotensin receptor blocker; CCB = calcium channel blocker; Diuretic included either hydrochlorothiazide or furosemide.

**Table 4:** Univariate relationship of AHI with covariates.

Covariates	β	P value
Age (years)	0.29	0.017
Male	0.27	0.023
Body mass index (kg/m^2^)	0.36	<0.01
Neck girth (cm)	0.40	<0.01
Current smoking, n (%)	0.09	0.167
eGFR (ml/min/1.73 m^2^)	0.11	0.119
Diabetes mellitus	0.07	0.264
PAC (ng/dL)	0.44	<0.01
PRA (ng/mL/h)	0.15	0.095
24 h-urine aldosterone (ug)	0.49	<0.01
Diuretic	−0.20	0.106
Spironolactone	−0.47	<0.01
ACEI/ARB	−0.06	0.195

Denote: eGFR = estimated glomerular filtration rate; PAC = plasma aldosterone concentration; PRA = plasma renin activity; ARR = aldosterone-renin ratio; ACEI = angiotensin converting enzyme inhibitor; ARB = angiotensin receptor blocker.

**Table 5:** Multivariate relationship of AHI with covariates.

Covariates	Model 1(β)	Model 2 (β)	Model 3 (β)
Age (years)	0.14^&^	NS	NS
Male	0.11^&^	NS	NS
Body mass index (kg/m^2^)	0.29*	0.16^&^	NS
Neck girth (cm)	0.35*	0.29*	0.23*
PAC (ng/dL)	0.43^#^	0.40^#^	0.32*
24 h-urine aldosterone (ug)	0.45^#^	0.42^#^	0.35*
Spironolactone	−0.42^#^	−0.38^#^	−0.30*

Denote: ^&^P > 0.05; *P < 0.05; ^#^P < 0.01; NS = non-significant; PAC = plasma aldosterone concentration; Model 1 adjusted for age and male gender; Model 2 additionally adjusted for body mass index and neck girth; Model 3 additionally adjusted for PAC, 24 h-urine aldosterone and spironolactone.
